# Perspectives of Patients Seeking Bariatric Surgery: The Impact of Early Patient-Provider Communication on Bariatric Surgery Utilization

**DOI:** 10.1007/s11695-025-08305-6

**Published:** 2025-10-12

**Authors:** Nitin Srinivasan, Jamil Samaan, Nithya Rajeev, Agnes Premkumar, Kelvin Alvarez, Lucy Harvey, Stephanie Nguyen, Ashley Tran, Kamran Samakar

**Affiliations:** 1 University of Southern California, Los Angeles, United States; 2 Cedars-Sinai Medical Center, Los Angeles, United States; 3 Creighton University Medical Center, Omaha, United States

**Keywords:** Bariatric surgery, Obesity stigma, Patient perception

## Abstract

**Introduction:**

Bariatric surgery (BS) is an effective and durable long-term treatment for severe obesity, yet less than 1% of eligible patients undergo surgery. Thus, we explored the perceptions and experiences of patients with obesity seeking BS prior to obtaining a referral for surgery.

**Methods:**

A survey was prospectively administered to patients who were referred to our institution from March 25, 2023, to May 28, 2024. Questions assessed patients' initial exposure to BS, perceptions of its safety and efficacy, who initiated referral discussions, and whether patients would have considered surgery earlier if prompted by their healthcare provider. Statistical analyses included Chi-square test. Qualitative analysis using thematic analysis was conducted for free-text responses.

**Results:**

Out of 327 patients invited, 118 completed the survey (36.1%). The majority of participants (57.9%) initiated discussions about BS with their provider, and 79.7% would have considered BS sooner if approached earlier. Respondents had considered BS for 3.8 years on average (standard deviation = 5.3). The most important contributions of BS for patients were “improved health” (94.1%) and “improved appearance” (31.6%). The most commonly reported barriers to obtaining a referral included cost (55.1%), followed by safety (45.8%) and efficacy (33.1%) concerns.

**Discussion:**

Our findings underscore the importance of proactive and early engagement between providers and patients regarding BS referrals. Addressing safety and efficacy concerns through comprehensive patient education by providers may improve BS referral rates by removing patients’ perceived barriers to care. Financial concerns remain a significant barrier to BS utilization and should be further investigated and addressed.

**Supplementary Information:**

The online version contains supplementary material available at 10.1007/s11695-025-08305-6.

## Introduction/purpose

Obesity poses a significant public health challenge, contributing to the burden of various chronic conditions such as cardiovascular disease, diabetes, hypertension, and malignancy [1]. Over the past 20 years, the prevalence of obesity among American adults has significantly increased, now affecting over 40% of adults [2]. Obesity affects almost 1 billion people worldwide, having doubled in prevalence since 1990 [3]. Bariatric surgery (BS) is an effective evidence-based treatment option for obesity, facilitating sustained weight loss and reducing obesity-related comorbidities [4–7]. BS is safe, shown to have a less than 6% risk of 30-day adverse events and a comparable mortality rate to other common general surgery procedures [8, 9]. However, despite its demonstrated safety and efficacy, less than 1% of eligible patients undergo BS [10].

The underlying reasons for the underutilization of BS are complex and multifaceted, likely involving a combination of socioeconomic, systemic, and psychosocial factors [11, 12]. Some studies have suggested that patient perceptions may affect utilization rates, with many patients viewing BS as unsafe or risky [13]. Others have highlighted the role of healthcare providers, with many physicians demonstrating a lack of familiarity with the safety and efficacy profile of the procedure [11]. However, while there are multiple studies evaluating the experiences of providers in referring patients to BS, there are very few studies examining patients’ experiences with regards to obtaining a BS referral [12].

We aim to address this gap in the literature by surveying patients referred for BS at our institution and highlighting their experience regarding their referral. Through our study, we hope to explore patient perceptions and motivations for pursuing BS, identify barriers to BS from the patient perspective, and highlight opportunities to improve the BS referral process during patient-provider discussions.

## Materials and Methods

Surveys were developed in both English and Spanish (Supplementary Files 1 and 2). The Spanish version was translated by a native bilingual investigator (K.A.). All patients referred for BS with our tertiary care academic center were invited to participate prior to their initial consultation. Survey items were developed by three investigators (N.S., J.S., and A.P.) and were informed by the expert opinion of K.S., a bariatric surgeon. Survey content was also guided by a previously published patient survey on BS referral [14]. The survey collected demographic information and data on initial exposures to BS, conversations with providers about surgery, motivations for surgery, and perceptions of surgery. The survey included multiple-choice questions, Likert-scale questions, and free-text responses. Prior to the study, we conducted a pilot study to ensure survey clarity and consistency. The survey was launched on March 25, 2023, and all data through May 28, 2024 were analyzed.

### Statistical Analysis

Quantitative data are presented as counts, percentages, standard deviations (SD), and interquartile ranges (IQR). A p-value of < 0.05 was considered statistically significant for all analyses. Qualitative data from free-text responses were analyzed using thematic analysis [15]. One investigator (N.S.) initially reviewed all responses to develop themes, excluding irrelevant responses. Two investigators (N.S. and N.R.) subsequently coded all responses independently, resolving any discrepancies through discussion. Descriptive analyses were conducted by two investigators (N.S. and N.R.) to quantify the number of responses within each coded theme. Microsoft Excel (version 16.75) was used for all analyses.

## Results

### Respondents

Out of 327 patients who were emailed the survey link, 118 patients completed the survey, resulting in a 36.1% response rate. The majority (n = 117) completed the English version, and one completed the Spanish version. Most respondents were female (79.7%), with an average age of 45.6 years and an average BMI of 43.5. A total of 55.6% of respondents identified as Hispanic, 30.8% as White and 11.1% as Black. Most respondents (50.8%) were either married or in a partnership. Thirty-nine percent of respondents completed part of college, while 22.0% completed a doctoral or professional degree. The vast majority of respondents were employed (71.2%), and many had previously worked in the healthcare field (41.4%). The average weight of respondents was 119.3 kg, while the average ideal body weight after BS was 76.0 kg (Table 1).

**Table 1:** Respondent demographics (n = 118)

Age (years), mean ± SD		45.6 ± 11.7
Female, n/total (%)		94/118 (79.7)
English-speaking, n/total (%)		117/118 (99.2)
Race/Ethnicity, n/total (%)	American Indian or Alaska Native	3/117 (2.6)
	Asian	4/117 (3.4)
	Black/African American	13/117 (11.1)
	Hispanic	65/117 (55.6)
	Native Hawaiian or Other Pacific Islander	1/117 (0.9)
	White	36/117 (30.8)
	Other	2/117 (1.7)
Marital Status, n/total (%)	Single	40/118 (33.9)
	Married/In a partnership	60/118 (50.8)
	Widowed	3/118 (2.5)
	Divorced	14/118 (11.9)
	Separated	1/118 (0.8)
Highest Education Completed, n/total (%)	Part of high school	3/118 (2.5)
	High school diploma	23/118 (19.5)
	Part of college	46/118 (39.0)
	Bachelor’s degree	20/118 (16.9)
	Doctoral or professional degree	26/118 (22.0)
Employment Status, n/total (%)	Employed	84/118 (71.2)
	Unemployed	19/118 (16.1)
	Student	1/118 (0.8)
	Retired	14/118 (11.9)
	Previously worked in the healthcare field	48/116 (41.4)
Height (inches), mean ± SD, n		65.2 ± 3.6, 118
Weight (kg), mean ± SD, n		119.3 ± 24.8, 116
BMI (kg/m^2^), mean ± SD, n		43.5 ± 8.8, 118
Lifetime maximum weight (kg), mean ± SD, n		127.8 ± 27.6, 117
Ideal body weight (kg), mean ± SD, n		76.0 ± 13.7, 109
Ideal expected weight loss (kg), mean ± SD, n		43.4 ± 20.7, 108

Responses were incomplete for some questions, so the sample size (n) varies by category.

*BMI*, body mass index; *SD*, standard deviation.

#### Patient-Provider Communication

**Fig. 1:**
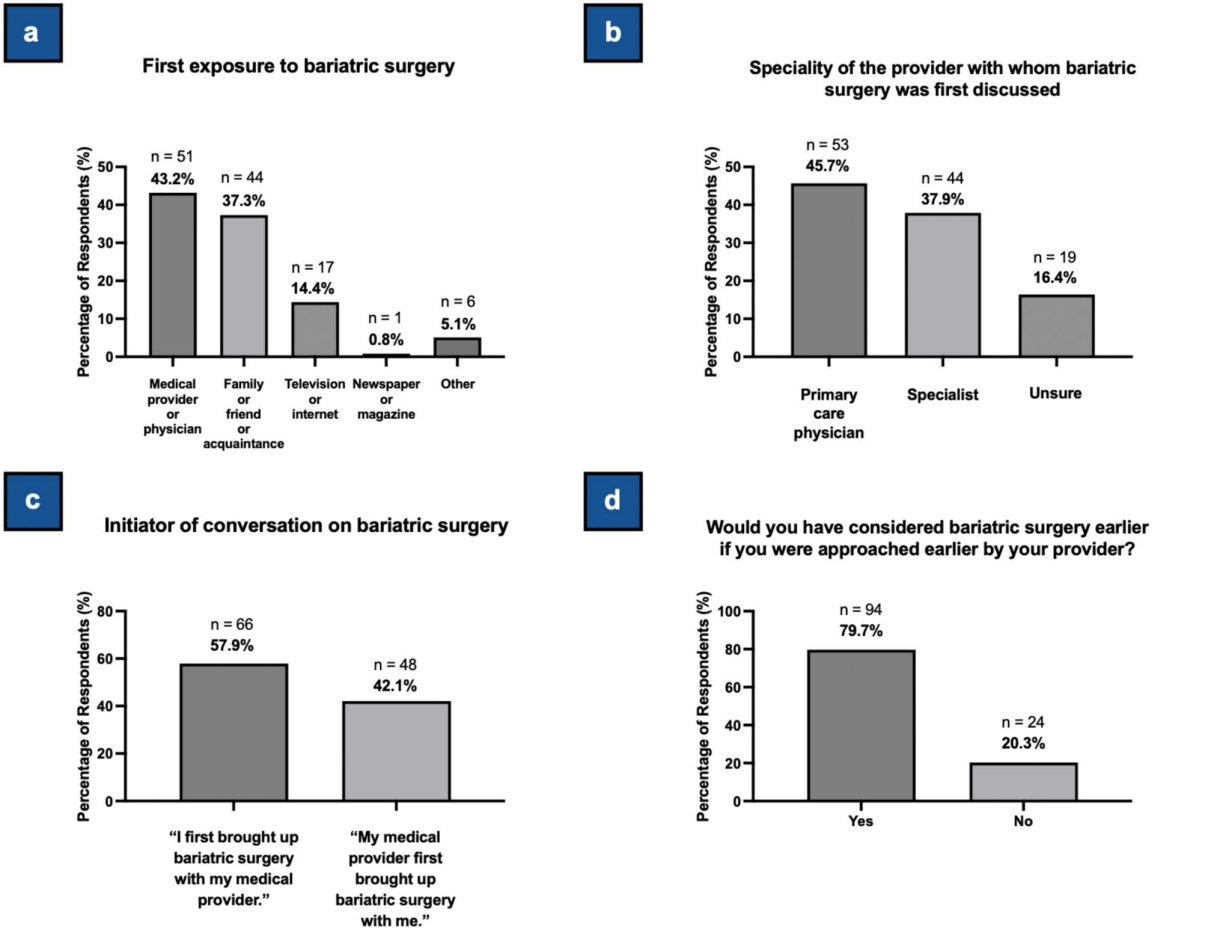
Survey Data on Patient-Provider Communication Regarding Bariatric Surgery. **1a***.* Survey data showing how respondents first learned about bariatric surgery. Percentages are based on a total of 118 respondents. One respondent indicated two answer choices, so the total number of responses is 119. Respondents selecting “Other” provided more details via free-text responses; select responses are shown in Table 2. **1b***.* Survey data showing the specialty of the provider with whom bariatric surgery was first discussed. Percentages are based on a total of 116 respondents (2 did not answer this question). **1c**. Survey data showing who initiated the conversation about bariatric surgery between the patient and their provider. Percentages are based on a total of 114 respondents (4 did not answer this question). **1d**. Survey data showing whether respondents would have considered bariatric surgery sooner if approached earlier by their providers. Percentages are based on a total of 118 respondents

The plurality of respondents (43.2%, n = 51) were introduced to BS by their healthcare provider, followed by family or friends (37.3%, n = 44) (Table 2, Fig. 1a). With respect to their initial conversation about BS, 45.7% of respondents first discussed BS with a primary care physician (n = 53), while 37.9% first discussed BS with a specialist (n = 44) (Table 2, Fig. 1b). The majority (57.9%, n = 66) initiated discussions about BS with their provider, while 42.1% (n = 48) reported that their provider initiated the conversation (Table 2, Fig. 1c). Most respondents (79.7%, n = 94) would have considered BS sooner if approached earlier (Table 2, Fig. 1d).

**Table 2:** Summary of patient responses to survey questions related to patient-provider communication

Question	No. of Respondents	Answers	N (%)	Example Response
How did you first hear or learn about BS/WLS?	118	Medical provider or physician	51 (43.2)	
		Friend or family or acquaintance	44 (37.3)	
		Television or internet	17 (14.4)	
		Newspaper or magazine	1 (0.8)	
		Other	6 (5.1)	
Please specify how you first heard about BS/WLS. (if answered “Other” to question above)^**a**^	6	At place of employment	2 (33.3)	“At work”—Participant 33
		Insurance	2 (33.3)	“Aetna”—Participant 116
		Other	2 (33.3**)**	–
What was the specialty of the provider with whom you first discussed BS/WLS?	116	Primary care physician	53 (45.7)	
		Specialist	44 (37.9)	
		Unsure	19 (16.4)	
Were you the one who first brought up BS/WLS with a medical provider, or did a medical provider first bring up BS/WLS with you?	114	“I first brought up BS/WLS with my medical provider.”	66 (57.9)	
		“My medical provider first brought up BS/WLS with me.”	48 (42.1)	
Would you have considered surgery earlier if you were approached earlier by your physician/provider?	118	Yes	94 (79.7)	
		No	24 (20.3)	

^**a**^Free-text response question

*BS*, bariatric surgery; *ESG*, endoscopic sleeve gastroplasty; *WLS*, weight loss surgery.

The average length of time spent considering BS (n = 110) was 3.8 years (SD = 5.3), with a median of 2 years (IQR: 1–5). Patients considering BS for 2 or more years were significantly more likely (89.2%) than those considering for less than 2 years (71.1%) to report that they would have pursued surgery sooner if approached earlier by their provider (χ2 = 5.87, p = 0.02).

#### Motivations and Barriers

**Fig. 2:**
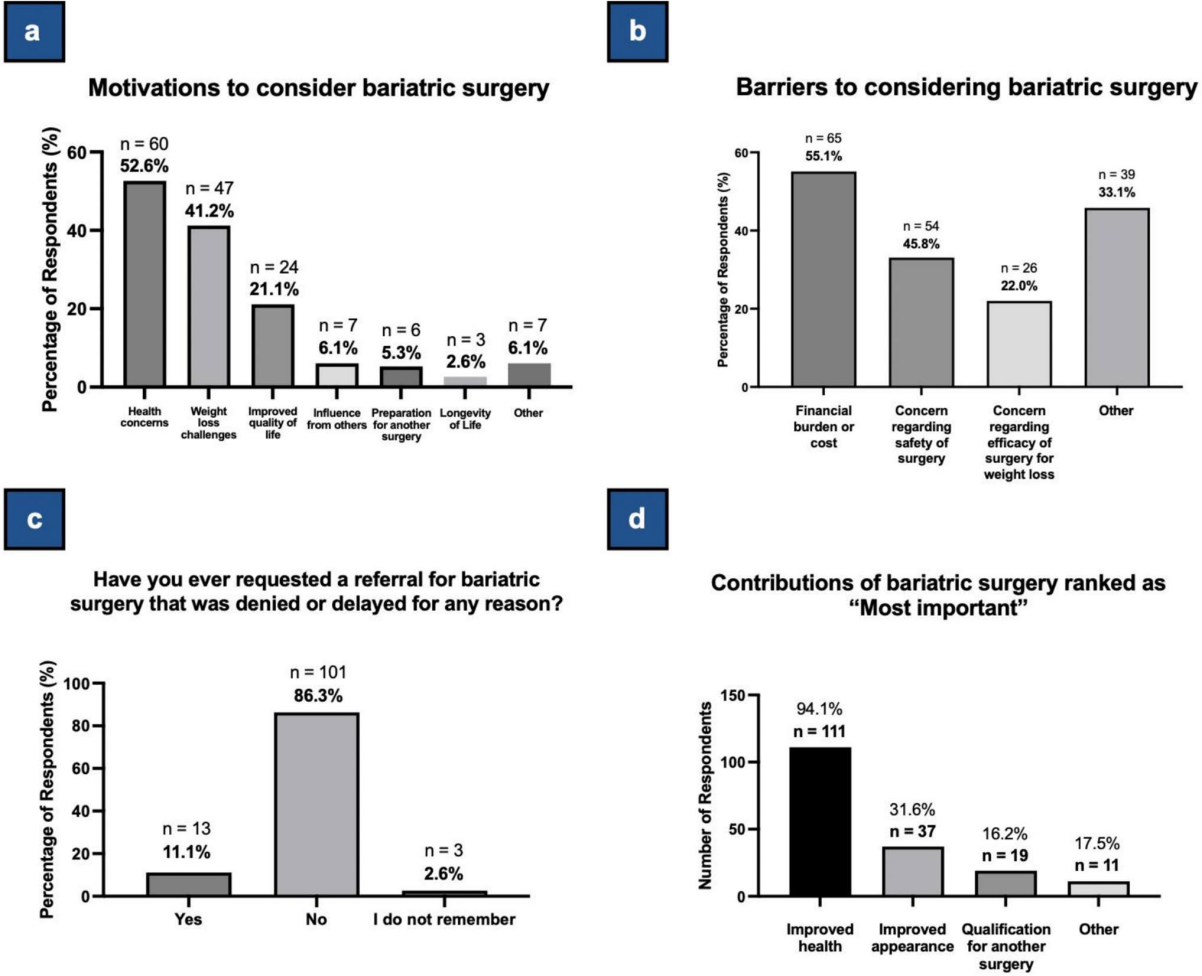
Survey Data on Motivations and Barriers Regarding Bariatric Surgery. **2a***.* Survey data showing respondents’ motivations for pursuing bariatric surgery. Responses were initially free-text and categorized by two researchers (N.S. and N.R.) into themes. Percentages are based on a total of 118 respondents. Data in tabular form is available in Table 3. **2b**. Survey data showing barriers to considering bariatric surgery. Percentages do not add up to 100% because respondents could select multiple answers. Percentages are based on a total of 118 respondents. Respondents selecting “Other” provided more details via free-text responses; select responses are shown in Table 3. **2c***.* Survey data showing whether respondents had ever requested a referral for bariatric surgery that was denied or delayed. Percentages are based on a total of 117 respondents (1 did not answer this question). Data in tabular form is available in Table 3. **2d***.* Survey data showing the number of respondents who marked various contributions of bariatric surgery as “Most important” on Likert-scale questions. Percentages are based on the number of respondents to each Likert-scale question: “Improved health” (118), “Improved appearance” (117), “Qualification for another surgery” (117), and “Other” (63). Respondents who selected “Other” provided more details via free-text responses; select responses are shown in Table 3

Free-text responses regarding motivations for considering BS were coded into six themes: health concerns (52.6%, n = 60), weight loss challenges (41.2%, n = 47), improved quality of life (21.1%, n = 24), influence from others (6.1%, n = 7), preparation for another surgery (5.3%, n = 6), and longevity of life (2.6%, n = 3) (Table 3, Fig. 2a). With respect to health concerns, respondents described various conditions such as hypertension, diabetes, hypercholesterolemia, fatty liver disease, arthritis, and depression as motivations to pursue BS. One respondent detailed a critical health incident: “I got sick and was taken to the hospital. I was told that if I did not have bariatric surgery ASAP, I would not make it another 6 months” (Participant 13). Additionally, several respondents expressed frustration over regaining lost weight and unsuccessful attempts with other weight loss programs. Examples included, “I keep going up and down in weight over the years. I need something to be more permanent,” (Participant 96) and “I lose weight, [then] hit a plateau, and no matter how hard I work at it, I stop making progress” (Participant 116). Furthermore, the desire for improved quality of life was a prevalent theme as well, with many respondents describing how their weight was causing significant discomfort and an inability to perform daily activities. One notable sub-theme was patients being motivated to improve health in order to increase engagement with family members, detailed in statements such as, “wanting to be in better health for my young grandkids” (Participant 40).

**Table 3:** Summary of patient responses to survey questions related to motivations and barriers

Question		No. of Responses	Answers/Topics	N (%)	Example Response (if applicable)
What motivated you to consider BS/WLS?^**a**^		114	Health concerns	60 (52.6)	“I got sick and was taken to the hospital. I was told that if I did not have bariatric surgery ASAP, I would not make it another 6 months. My weight is causing many medical issues; one of the major issues is my breathing”—Participant 13
			Weight loss challenges	47 (41.2)	“The difficulty I face losing weight and keeping it off after trying several things such as intermittent fasting, caloric deficit[s], workouts…”—Participant 2
			Improved quality of life	24 (21.1)	“I just want a better quality of life”—Participant 110
			Influence from others	7 (6.1)	“Seeing my sister go through this procedure…”—Participant 77
			Preparation for another surgery	6 (5.3)	"Need for kidney transplant…"—Participant 102
			Longevity of life	3 (2.6)	“Need to live longer”—Participant 25
			Other	7 (6.1)	
Prior to today, what were some barriers to you considering BS/WLS?		118	Financial burden or cost	65 (55.1)	
			Concern regarding safety of surgery	54 (45.8)	
			Concern regarding efficacy of surgery	39 (33.1)	
			Other	26 (22.0)	
Please specify any other barriers to you considering BS/WLS. (if answered “Other” to question above)^**a**^		19	Psychological or mental barriers	4 (21.1)	“Fear”—Participant 77
			Inability to accommodate dietary changes	3 (15.8)	“My ability to change the way I eat”—Participant 72
			Preference for alternative options	3 (15.8)	“I figured the best solution was [rather] to change my lifestyle and exercise”—Participant 111
			Concerns about pregnancy	2 (10.5)	“The need to wait a specific amount of time before getting pregnant [after surgery]”—Participant 2
			Chronic health conditions	2 (10.5)	“I have a handful of chronic conditions, and I want to ensure that I can continue to effectively manage them post surgery”—Participant 52
			Physical limitations	2 (10.5)	“Inability to walk”—Participant 45
			Life responsibilities	1 (5.3)	“I have young children and the recovery concerned me”—Participant 79
			Resistance to taking a perceived shortcut	1 (5.3)	"I didn't want to feel like it was the "easy way out" or like I was "lazy;" I wanted to improve on my own and only consider [surgery] once I had [exhausted all other options]”—Participant 80
			Other	1 (5.3)	–
Have you ever requested a referral for BS/WLS that was denied or delayed for any reason?		117	Yes	13 (11.1)	
			No	101 (86.3)	
			I do not remember	3 (2.6)	
If delayed or denied, why was [your] request for a referral for BS/WLS delayed or denied?^**a**^		11	Lack of insurance coverage	4 (36.4)	“Insurance denied”—Participant 41
			COVID-19	1 (9.1)	“COVID-19”—Participant 63
			Health conditions	1 (9.1)	“… I have my health problems”—Participant 5
			Lack of dietary compliance	1 (9.1)	“… I didn't stay on their diet all the time”—Participant 115
			Previous history of bariatric surgery	1 (9.1)	“Previously [bariatric surgery was] done”—Participant 82
			Reason unknown	1 (9.1)	“Unknown”—Participant 75
			Surgery not medically necessary	1 (9.1)	“ESG was not medically necessary”—Participant 47
			Weight gain	1 (9.1)	“Weight gain”—Participant 101
What do you believe are the major contributions of BS/WLS to your life? Rank the following from least to most important contribution as they relate to your decision to pursue BS/WLS	Improved health	118	1, Least important	2 (1.7)	
			2	0 (0.0)	
			3	2 (1.7)	
			4, Most important	111 (94.1)	
			Not applicable	3 (2.5)	
	Improved appearance	117	1, Least important	9 (7.7)	
			2	27 (23.1)	
			3	42 (35.9)	
			4, Most important	37 (31.6)	
			Not applicable	2 (1.7)	
	Qualification for another surgery	117	1, Least important	47 (40.2)	
			2	6 (5.1)	
			3	9 (7.7)	
			4, Most important	19 (16.2)	
			Not applicable	36 (30.8)	
	Other	63	1, Least important	15 (23.8)	
			2	2 (3.2)	
			3	4 (6.3)	
			4, Most important	11 (17.5)	
			Not applicable	31 (49.2)	
Do you believe BS/WLS provides you any other contributions, apart from those mentioned above? If so, please specify. (if answered “Other” to question above)^**a**^		36	Improved quality of life	18 (50.0)	“Quality of my life would be better”—Participant 42
			Increased activity and mobility	11 (30.6)	“The ability to be as active as I desire to be”—Participant 78
			Improved mental health	4 (11.1)	“Mental health improvement, leading to life improvements”—Participant 104
			Improved life expectancy	3 (8.3)	“I want to be around for my daughters, so this is about the longevity of [my] life”—Participant 37
			Preparation for surgery	3 (8.3)	"… I [can later get] bilateral knee surgery"—Participant 87
			Improved diet	2 (5.6)	“Improve[d] eating habits”—Participant 19
			Other	5 (13.9)	–

^**a**^Free-text response question

*BS*, bariatric surgery; *WLS*, weight loss surgery.

Cost was the most common barrier to considering BS (55.1%, n = 65), followed by concerns regarding safety (45.8%, n = 54) and efficacy (33.1%, n = 39) (Table 3, Fig. 2b). Among those who selected “Other” (22.0%, n = 26), psychological barriers (21.1%, n = 4) was the most common theme cited in free-text responses (n = 20) (Table 3). Respondents explicitly mentioned a “fear” of surgery (Participant 77), lack of “mental preparation” (Participant 90), as well as feeling judged by others for pursuing BS (Participant 37).

A small subset of respondents (11.1%, n = 13) reported having experienced a delay or denial in referral for BS (Fig. 2c, Table 3). Among free-text responses (n = 11), lack of insurance coverage (n = 4, 36.4%) was the most cited reason for delay or denial (Table 3).

Among the various expected major contributions of BS, “improved health” (94.1%, n = 111) was most frequently ranked as “most important” on the Likert scale, followed by “improved appearance” (31.6%, n = 37) and “qualifying for another surgery” (16.2%, n = 19) (Table 2, Fig. 2d). Among all respondents who selected “Other” (n = 63), “improved quality of life” (50.0%, n = 18) and “increased activity and mobility” (30.6%, n = 11) were the most common contributions cited in free-text responses (n = 36) (Table 3). Within the quality of life theme, respondents often emphasized their desire to re-engage with family. One respondent expressed: “…I want to do the things with my family that we used to do. I want to be at all my grandkids’ events.” (Participant 13). The desire to improve activity and mobility was also prevalent, with one patient having the general aspiration of “[becoming] more active,” (Participant 17) while another expressing the specific goal of “being able to walk again” (Participant 57).

#### Perceptions

**Fig. 3:**
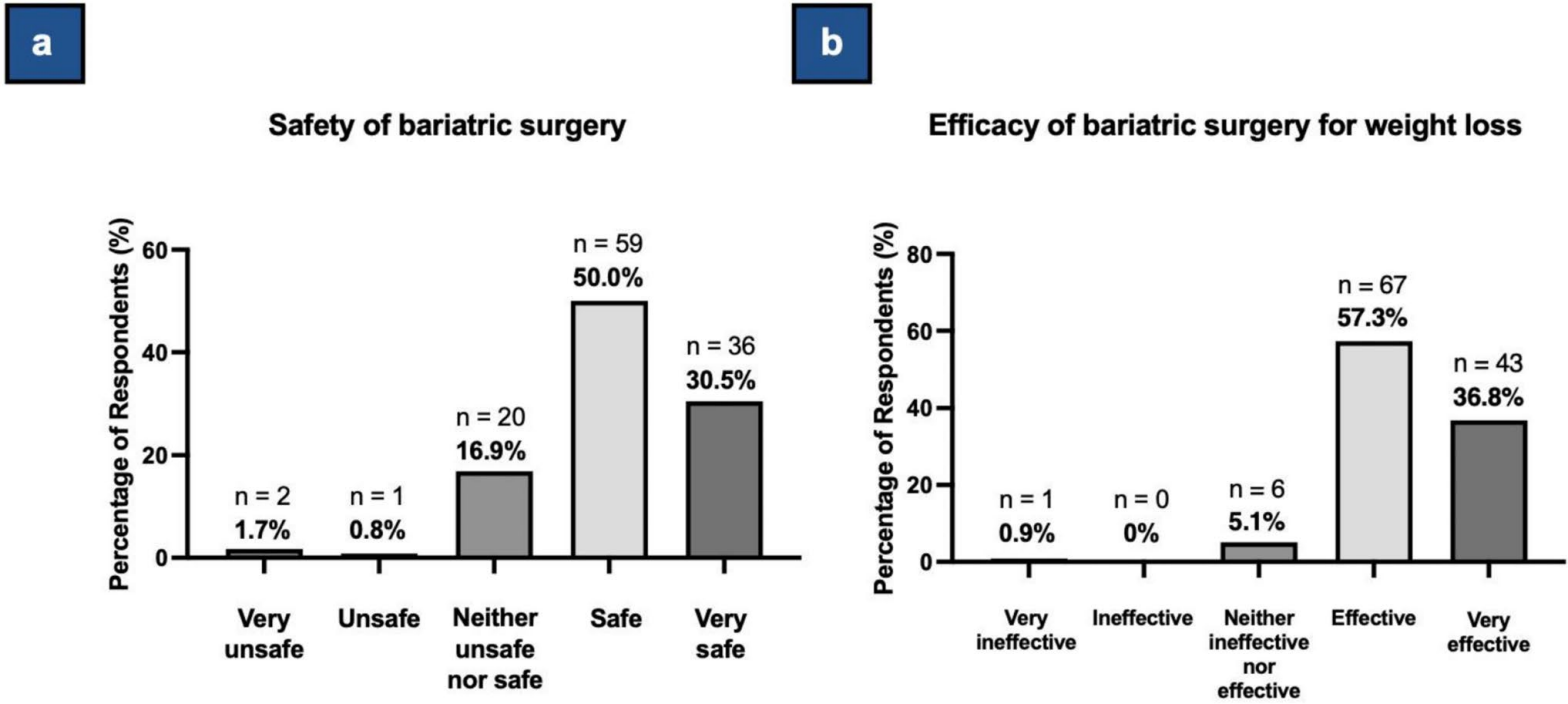
Survey Data on Perceptions Regarding Bariatric Surgery. **3a**. Survey data showing respondents’ perceptions of the safety of bariatric surgery. Percentages are based on a total of 118 respondents. **3b**. Survey data showing respondents’ perceptions of the efficacy of bariatric surgery for significant, long-term weight loss. Percentages are based on a total of 117 respondents (1 did not answer this question)

Most respondents believe that BS is safe (50.0%, n = 59) or very safe (30.5%, n = 36) (Table 4, Fig. 3a). Additionally, most respondents perceive BS to be effective (57.3%, n = 67) or very effective (36.8%, n = 43) for significant, long-term weight loss (Table 4, Fig. 3b).

**Table 4:** Summary of patient responses to survey questions related to perceptions

Question	No. of Respondents	Answers	N (%)
*Q23*, How safe do you think BS/WLS is?	118	Very unsafe	2 (1.7)
		Unsafe	1 (0.8)
		Neither unsafe nor safe	20 (16.9)
		Safe	59 (50.0)
		Very safe	36 (30.5)
*Q24,* How effective do you think BS/WLS is for significant, long-term weight loss?	117	Very ineffective	1 (0.9)
		Ineffective	0 (0.0)
		Neither ineffective nor effective	6 (5.1)
		Effective	67 (57.3)
		Very effective	43 (36.8)

## Discussion

We aimed to explore the experiences and perceptions of patients with obesity seeking BS prior to obtaining a referral for surgery. Most (57.9%) respondents initiated the first conversation about BS with their healthcare provider and the majority (79.7%) reported that they would consider surgery sooner if approached earlier by their provider. Longer length of time of considering BS was associated with a higher likelihood of considering surgery sooner if approached earlier. Lastly, commonly reported barriers by patients included financial cost, followed by concerns regarding safety and efficacy. Overall, our study underscores the importance of early communication between patients and referring providers regarding BS referrals.

Our findings highlight the critical need for providers to initiate early, comprehensive discussions about BS with eligible patients. Notably, less than half of our participants reported that their provider initiated a conversation about BS during clinic visits. This aligns with prior research by Huq et al. (2020) [16] and Primomo et al. (2016) [14], which identified low rates of provider-initiated discussions about BS. Furthermore, most respondents indicated that they would have considered BS earlier had it been introduced sooner by their provider. Interestingly, a longer length of time considering BS was associated with a higher likelihood of considering surgery earlier if providers had introduced it sooner. This suggests that, for some patients, the primary barrier to pursuing surgery was simply the absence of a discussion initiated by their provider. These findings underscore the value of initiating conversations early, allowing patients more time to carefully weigh the risks and benefits, explore BS as an option compared to other treatments, and make informed decisions [17, 18]. Early discussions could also facilitate timelier surgeries, thereby increasing the utilization of BS and improving patient outcomes. This is consistent with prior studies that have shown that earlier surgeries lead to more significant reductions in obesity-related health conditions compared to delayed interventions [19, 20].

Despite previous research showing that providers generally have positive attitudes toward BS and understand its benefits, physicians remain reluctant to refer patients or initiate discussions about surgery [14, 21, 22]. Contributing factors include unfamiliarity with referral guidelines, concerns about risks and complications, and discomfort with post-operative care [23–26]. Addressing these issues through better education for physicians on American Society for Metabolic and Bariatric Surgery referral criteria, perioperative risks, and postoperative management could help mitigate resistance among providers [7, 25]. Notably, our finding that 16.2% of respondents sought BS to qualify for another procedure suggests that surgeons in other specialties may serve as valuable partners in identifying candidates and educating patients about BS. Targeted education efforts to engage these providers could foster multidisciplinary collaboration and promote earlier patient engagement with surgery.

Financial considerations also contribute to providers’ reluctance towards BS referral. Our survey identified financial burden as the greatest barrier for patients considering BS. Well-documented financial obstacles include the cost of surgery, patient perceptions regarding its affordability, and uncertainty about insurance coverage [23, 25–28]. While patients often view BS as expensive, the specific factors shaping these perceptions remain largely unexplored in the literature. These perceptions may be influenced by various factors, such as differences in insurance coverage policies, the financial practices of bariatric programs, and additional costs associated with the surgery (e.g. lost wages, childcare, protein supplements). For example, one study found that commercially insured patients incurred annual out-of-pocket costs ranging from $1,083 to $1,266 after sleeve gastrectomy and from $1,228 to $1,377 after Roux-en-Y gastric bypass during the first three postoperative years [29]. Furthermore, while some private and community-based programs require patients to pay $2,500 to $10,000 beyond insurance coverage, many academic programs do not impose these fees, contributing to disparities in access depending on where patients receive care. Unfortunately, detailed and transparent cost data across programs remain limited, complicating efforts to address financial barriers.

Despite these financial barriers, BS has been shown to be cost-effective compared to the medical management of morbid obesity and its related comorbidities within as little as two years [30–33]. While further studies are needed to better understand patient perspectives on the financial burdens of BS, increased public funding to cover the short-term costs of surgery may be helpful in improving access to BS and may increase provider comfort with making referrals.

Additionally, our study underscores the importance of improved communication between patients and providers. While most patients perceived BS as safe and effective, concerns about safety and efficacy were still prominent barriers. This indicates that positive attitudes and general knowledge alone may not suffice to improve utilization rates. Providers should engage in communication strategies tailored to individual patients’ concerns and knowledge gaps to maximize BS utilization [34]. Shared decision-making is considered the gold standard of patient-centered care and should form the basis of all patient-provider discussions regarding BS [35]. Other strategies include the creation of patient education materials that help patients compare the risks and benefits of different treatment options. In the context of BS, the use of patient education materials is associated with postoperative patient satisfaction and decreased postoperative regret [36].

Beyond traditional brochures, lectures, and virtual written materials, the growing demand for personalized and accessible health information points to the need for more innovative educational tools. Our finding that 14% of patients first learned about BS through television or the internet reflects how digital media has become a routine part of health knowledge acquisition. However, while these platforms offer broad access, they often lack personalization, interactivity, and clinical integration. This creates an opportunity to explore emerging technologies that can deliver more tailored, accurate, and accessible patient education across diverse settings, including clinical environments. These emerging tools are not limited to web-based platforms; they can be integrated into mobile apps, embedded in clinical decision support systems, or used during provider-patient interactions to facilitate tailored education in real time. Among the most promising of these innovations is the use of artificial intelligence (AI), particularly large language models (LLMs) like Chat Generative Pretrained Transformer (ChatGPT). LLMs have demonstrated the ability to deliver accurate, comprehensive, and reliable health information across a variety of specialties, including cardiology, gastroenterology, nephrology, hepatology, as well as bariatric surgery [37–43]. Multiple studies have shown the accuracy, comprehensiveness, and reliability of responses by LLMs to questions related to BS [38, 44–47]. One study also demonstrated that ChatGPT can enhance the readability of patient education materials when appropriately prompted [48]. These findings highlight the potential for developing LLM-powered applications that provide dynamic, engaging, accurate, reliable, and easy to understand patient education materials to patients of all health literacy levels. If validated in prospective studies, these tools can help bridge the education gap for patients considering BS and thereby increase the utilization of BS.

This study has several limitations, including a small sample size and potential selection bias, as only patients willing to complete the survey participated. The reliance on self-reported data introduces risks of recall and social desirability biases. Thematic analysis of free-text responses may also be prone to subjectivity and bias in interpretation. Notably, 41% of respondents reported working in healthcare-related fields, which may have introduced bias. These individuals might be more receptive to BS as a treatment option, more likely to initiate discussions with their providers, or more inclined to trust medical advice. As such, their responses may not fully reflect the perceptions and barriers experienced by the general population. Furthermore, the study was conducted at a single institution, which may also limit generalizability.

In conclusion, our study highlights the patient experience regarding obtaining a BS referral, as well as patient perceptions and barriers to utilization. Our findings underscore the necessity of provider engagement in comprehensive communication with patients to address any concerns about risks, complications, and long-term efficacy. Initiating conversations about BS at an earlier stage can result in earlier BS utilization and improved patient outcomes. Additionally, improving physician education on referral criteria, postoperative management, and the safety and efficacy of BS may reduce barriers to referral. Further studies on patient perceptions regarding the financial barriers of BS are also warranted. Lastly, we encourage future investigations exploring the efficacy, safety, development, and validation of AI-based applications for bridging the education gap for patients considering BS. By improving patient-provider communication, addressing barriers, and improving the delivery of patient education materials, we can enhance the utilization and outcomes of BS, ultimately benefiting patients' health and quality of life.

## Supplementary Information

Below is the link to the electronic supplementary material.

ESM 1 (PDF 1.07 MB)

ESM 2 (PDF 1.15 MB)

## Data Availability

All data supporting the findings of this study are available from the corresponding author upon reasonable request.
