# Supplementary material for: Perspectives of Patients Seeking Bariatric Surgery: The Impact of Early Patient-Provider Communication on Bariatric Surgery Utilization
Source: Obes Surg. 2025 Oct 12;35(11):4639–51. doi: 10.1007/s11695-025-08305-6 (PMC12594654; doi:10.1007/s11695-025-08305-6)
Supplement: Supplementary file 1 — (PDF 1.07 MB) [file 11695_2025_8305_MOESM1_ESM.pdf]

Perceptions of Bariatric Surgery Seminar Participants

Please complete the survey below prior to viewing the bariatric seminar materials.

Thank you!

You are asked to participate in an anonymous patient survey conducted by Dr. Kamran Samakar, Director of Bariatric Surgery at Keck Medical Center. This study is being done to better understand the experience of bariatric surgery candidates prior to obtaining a referral. This survey will take approximately 10 minutes. We hope that the results of this study will be used to improve access and outcomes for bariatric surgery patients in the future. There is no cost to you for taking part in this study. Your participation in this study is entirely voluntary. Your answers to questions will not impact your care in any way. Please do not answer any questions that you are uncomfortable answering and you may choose to stop filling out the survey at any time. Thank you very much for considering participating in our study.

---

1 What is your age (in years)?

____

2 Current date (MM-DD-YYYY)

____

3 What is your sex?

- ◯ Female
- ◯ Male
- ◯ Other
- ◯ Prefer not to answer

4 Please specify your sex.

____

5 Which of the following best describes you? (Check all that apply)

- ☐ American Indian or Alaska Native
- ☐ Asian
- ☐ Black or African American
- ☐ Hispanic
- ☐ Native Hawaiian or Other Pacific Islander
- ☐ White
- ☐ Other

6 Please specify your race/ethnicity.

____

7 What is your marital status?

- ◯ Single (never married)
- ◯ Married, or in a domestic partnership
- ◯ Widowed
- ◯ Divorced
- ◯ Separated

8 What is the highest degree or level of education you have completed?

- ◯ Some high school
- ◯ High school diploma
- ◯ Some college
- ◯ Bachelors
- ◯ Doctoral or professional degree

9 What is your current employment status?

- ◯ Employed
- ◯ Unemployed
- ◯ Student
- ◯ Retired

10 Do you currently, or have previously in the past, worked in the healthcare field?

- ◯ Yes
- ◯ No

11 What is your current height? (in feet and inches- ex. 5 feet 3 inches)

____

12 What is your current weight? (in pounds)

____

13 What is the highest weight you have ever been? (in pounds)

____

14 How did you first hear or learn about bariatric surgery/weight loss surgery?

- ◯ Medical provider/Doctor
- ◯ Family/ Friend/ acquaintance
- ◯ Television/internet
- ◯ Newspaper/Magazine
- ◯ Other

15 Please specify how you first heard about bariatric surgery/weight loss surgery.

____

16 What was the speciality of the provider with whom you first discussed bariatric surgery/weight loss surgery?

- ◯ Primary care doctor (Family Medicine doctor, Generalist, Internal Medicine)
- ◯ Specialist
- ◯ Unsure

17 Were you the one who first brought up bariatric surgery/weight loss surgery with a medical provider, or did a medical provider first bring up bariatric surgery/weight loss surgery with you?

- ◯ I first brought up bariatric surgery/weight loss surgery with my medical provider.
- ◯ My medical provider first brought up bariatric surgery/weight loss surgery with me.

18 How long have you been considering bariatric surgery/weight loss surgery?

____

19 What motivated you to consider bariatric surgery/weight loss surgery?

____

20 Would you have considered surgery earlier if you were approached earlier by your physician/provider?

- ◯ Yes
- ◯ No

21 Have you ever requested a referral for bariatric surgery/weight loss surgery that was denied or delayed for any reason?

- ◯ Yes
- ◯ No
- ◯ I do not remember

22 Why was this request for a referral for bariatric surgery/weight loss surgery delayed or denied?

____

23 How safe do you think bariatric surgery/weight loss surgery is?

- ◯ Very safe
- ◯ Safe
- ◯ Neither safe nor unsafe
- ◯ Unsafe
- ◯ Very unsafe

24 Please rate how effective you think bariatric surgery/weight loss surgery is for significant, long-term weight loss?

- ◯ Very effective
- ◯ Effective
- ◯ Neither effective nor ineffective
- ◯ Ineffective
- ◯ Very ineffective

What do you believe are the major contributions of bariatric surgery/weight loss surgery to your life? Rank the following from least to most important contribution as they related to your decision to pursue bariatric surgery/weight loss surgery.

		Least Important			Most Important	Not applicable
25	Improved health	○	○	○	○	○
26	Improved appearance	○	○	○	○	○
27	It would allow me to qualify for another surgery	○	○	○	○	○
28	Other	○	○	○	○	○

29 Do you believe bariatric surgery/weight loss surgery provides you any other contributions, apart from those mentioned above? If so, please specify. ________________________________

30 Prior to today, what were some barriers to you considering bariatric surgery/weight loss surgery? Mark all that apply.

- ☐ Safety of surgery
- ☐ Effectiveness of surgery for weight loss
- ☐ Financial/cost
- ☐ Other

31 Please specify any other barriers to you considering bariatric surgery/weight loss surgery. ________________________________

32 After you have undergone bariatric surgery/weight loss surgery, what would be your ideal weight (in pounds)? ________________________________
