# Supplementary material for: Perspectives of Patients Seeking Bariatric Surgery: The Impact of Early Patient-Provider Communication on Bariatric Surgery Utilization
Source: Obes Surg. 2025 Oct 12;35(11):4639–51. doi: 10.1007/s11695-025-08305-6 (PMC12594654; doi:10.1007/s11695-025-08305-6)
Supplement: Supplementary file 2 — (PDF 1.15 MB) [file 11695_2025_8305_MOESM2_ESM.pdf]

Percepciones de los participantes del seminario de cirugía bariátrica en español

Complete la siguiente encuesta antes de ver los materiales del seminario bariátrico.

¡Gracias!

Se le ha pedido participar en un encuesta anónima de pacientes dirigida por el Dr. Kamran Samakar, el Director de Cirugía Bariátrica en el Keck Medical Center. Esta investigación se está realizando para ayudar a aumentar nuestro entendimiento de las experiencias de los candidatos de la cirugía bariátrica antes de recibir una referencia médica. La encuesta dura aproximadamente 10 minutos. Esperamos que los resultados de este estudio se utilicen para mejorar el acceso y los resultados de los pacientes de cirugía bariátrica en el futuro. No hay ningún costo para usted por participar en este estudio. La participación en el estudio es completamente voluntaria. Sus respuestas a las preguntas no afectarán su atención médica de ninguna manera. Por favor no conteste ninguna pregunta que le haga sentir incómodo y puede optar por dejar de completar la encuesta en cualquier momento. Muchas gracias por considerar participar en nuestro estudio.

---

1 ¿Cuál es su edad (años)?

____

---

2 Fecha actual (MM-DD-AAAA)

____

---

3 ¿Cual es su sexo?

- ◯ Masculino
- ◯ Femenino
- ◯ Otro
- ◯ Prefiero no responder

---

4 Por favor, especifique su sexo.

____

---

5 ¿Cuál de las siguientes lo/la describe mejor? (Marque todos los que le correspondan)

- ☐ Indio americano o nativo de Alaska
- ☐ Asiático
- ☐ Negro o afroamericano
- ☐ Hispano/Latino
- ☐ Nativo de Hawai o otra isla del Pacífico
- ☐ Blanco
- ☐ Otro

---

6 Por favor, especifique su raza/origen étnico.

____

---

7 ¿Cuál es su estado civil?

- ◯ Soltero (nunca casado)
- ◯ Casado o viviendo con su pareja
- ◯ Viudo
- ◯ Divorciado
- ◯ Separado

---

8 ¿Cuál es el grado o nivel de educación más alto que ha completado?

- ◯ Algun grado de primaria o secundaria
- ◯ Título de preparatoria
- ◯ Universidad empezada
- ◯ Licenciatura
- ◯ Doctorado

---

9 ¿Cuál es su situación laboral actual?

- ◯ Empleado
- ◯ Desempleado
- ◯ Estudiante
- ◯ Retirado

10	¿Actualmente trabaja o ha trabajado anteriormente en el area de salud?	◯ Sí<br>◯ No
11	¿Cuál es su altura actual? (en pies y pulgadas- ej. cinco pies y tres pulgadas)	__________________
12	¿Cual es su peso actual? (en libras)	__________________
13	¿Cuál es el peso más alto que ha tenido? (en libras)	__________________
14	¿Cómo escuchó o se enteró por primera vez acerca de la cirugía bariátrica/cirugía de pérdida de peso?	◯ Proveedor médico/médico<br>◯ Familiar/amigo/conocido<br>◯ Televisión/internet<br>◯ Periódico/revista<br>◯ Otro
15	Especifique cómo se enteró por primera vez de la bariátrica cirugía/cirugía de pérdida de peso.	__________________
16	¿Cuál era la especialidad del proveedor con el que habló por primera vez sobre la cirugía bariátrica/cirugía para perder peso?	◯ Médico de atención primaria (médico de medicina familiar, generalista, medicina interna)<br>◯ Especialista<br>◯ No lo sé
17	¿Fuiste tú el primero en hablar sobre la cirugía bariátrica/cirugía para perder peso con un proveedor médico, o fue un proveedor médico el primero en hablar contigo sobre la cirugía bariátrica/cirugía para perder peso?	◯ Yo mencioné primero la cirugía bariátrica/pérdida de peso cirugía con mi proveedor medico.<br>◯ Mi proveedor medico me mencionó por primera vez la cirugía bariátrica/cirugía de pérdida de peso.
18	¿Cuánto tiempo ha estado considerando la cirugía bariátrica/cirugía para bajar de peso?	__________________
19	¿Qué lo motivó a considerar la cirugía bariátrica/cirugía para bajar de peso?	______________________
20	¿Habría considerado la cirugía antes si su médico/proveedor lo hubiera contactado antes?	◯ Sí<br>◯ No
21	¿Alguna vez ha solicitado un pase para cirugía bariátrica/cirugía de pérdida de peso que fue denegada o retrasada por algún motivo?	◯ Sí<br>◯ No<br>◯ No me acuerdo
22	¿Por qué esta solicitud de derivación para cirugía bariátrica/cirugía de pérdida de peso retrasada o negada?	__________________
23	¿Qué tan segura cree que es la cirugía bariátrica/cirugía de pérdida de peso?	◯ Muy segura<br>◯ Segura<br>◯ No segura ni riesgosa<br>◯ Riesgosa<br>◯ Muy riesgosa

24 Califique qué tan efectiva cree que es la cirugía bariátrica/cirugía de pérdida de peso para una pérdida de peso significativa a largo plazo.

- ◯ Muy efectiva
- ◯ Efectiva
- ◯ Ni efectiva ni inefectiva
- ◯ Inefectiva
- ◯ Muy inefectiva

¿Cuáles cree que son las principales contribuciones de la cirugía bariátrica/cirugía de pérdida de peso a su vida? Clasifique las siguientes contribuciones de menor a mayor en relación con su decisión de someterse a una cirugía bariátrica/cirugía para perder peso.

	Menos importante			Lo más importante	No aplica
25 Mejorar la salud	◯	◯	◯	◯	◯
26 Mejorar la apariencia	◯	◯	◯	◯	◯
27 Calificar para otra cirugia	◯	◯	◯	◯	◯
28 Otro	◯	◯	◯	◯	◯

29 ¿Cree que la cirugía bariátrica/cirugía de pérdida de peso le brinda otras contribuciones, además de las mencionadas anteriormente? Si es así, por favor especifique.

____

30 Antes de hoy, ¿cuáles fueron algunas de las barreras para considerar la cirugía bariátrica/cirugía para perder peso? Marque todo lo que corresponda.

- ☐ Seguridad de la cirugía
- ☐ Efectividad de la cirugía para bajar de peso
- ☐ Costo financiero
- ☐ Otro

31 Por favor, especifique cualquier otra barrera para usted considerando cirugía bariátrica/cirugía de pérdida de peso.

____

32 Después de someterse a una cirugía bariátrica/pérdida de peso cirugía, ¿cuál sería su peso ideal (en libras)?

____
